# Raynaud’s Phenomenon After Fasciectomy for Dupuytren’s Contracture: A Case Report

**DOI:** 10.7759/cureus.79316

**Published:** 2025-02-19

**Authors:** Arpita Devashetty, Alex Nicholls

**Affiliations:** 1 Orthopaedics, Basingstoke and North Hampshire Hospital, Basingstoke, GBR

**Keywords:** dupuytren's contracture, dupuytren's fasciectomy, fasciectomy, raynaud’s phenomenon, vibration white finger

## Abstract

Open fasciectomy is a widely accepted treatment for Dupuytren’s contracture, with known complications such as vascular and nerve injury, stiffness, and recurrence. However, delayed vascular complications beyond these known risks remain largely unexplored.

A 57-year-old nonsmoking man underwent open fasciectomy for Dupuytren’s contracture of the little finger. Six months postoperatively, he developed Raynaud’s phenomenon in the operated finger, characterized by cold-induced pallor that resolved with rewarming. Examination revealed sluggish circulation in the ulnar digital artery and altered sensation on the ulnar side of the digit. Conservative management with cold avoidance effectively prevented the recurrence of symptoms.

This is the first reported case of Raynaud’s phenomenon following fasciectomy for Dupuytren’s contracture. Surgeons should be aware of this potential delayed complication, particularly in patients without traditional risk factors. Early recognition and patient education on cold avoidance can mitigate symptoms and improve postoperative outcomes.

## Introduction

Dupuytren’s disease manifests as a persistent and progressive fibroproliferative condition that affects the palmar fascia, resulting in an intractable and progressively debilitating flexion contracture of the fingers [1]. Open fasciectomy is a widely accepted and effective treatment for Dupuytren’s contracture, although it has been associated with complication rates of up to 26% [2]. Early complications include arterial or nerve injury, infection, skin necrosis, and wound dehiscence, while delayed complications may involve stiffness, recurrence, pseudoaneurysm, and inclusion cysts [3,4]. To date, no cases of Raynaud’s phenomenon following fasciectomy have been reported in the literature.

Raynaud’s phenomenon is characterized by vasospasms of the arteries in the extremities, often triggered by cold exposure [5]. It is classified into primary Raynaud’s phenomenon, which occurs without an underlying disease, and secondary Raynaud’s phenomenon, which is associated with conditions such as autoimmune diseases, vascular disorders, and repetitive hand trauma [5]. Additional risk factors include smoking, certain medications (e.g., beta-blockers and chemotherapy agents), exposure to vibrating tools, and occupational hazards leading to microvascular damage [5,6]. Research has linked Raynaud’s to repeated hand injuries, particularly those caused by occupational hazards such as the use of vibrational tools, which can lead to vascular damage over time [6]. However, it has not been described as a postoperative complication.

## Case presentation

A 57-year-old Caucasian man presented with a flexion contracture in the proximal interphalangeal joint (PIPJ) of his left little finger, gradually developing over one year without prior injury. He is a nonsmoker and works as an IT professional.

Examination revealed a palpable pre-tendinous cord in the left little finger, causing a 31° contracture at the PIPJ, measured with a goniometer. There was no involvement of the metacarpophalangeal (MP) or distal interphalangeal (DIP) joints, and he retained full active flexion. Houston’s tabletop test, which assesses the severity of Dupuytren’s contracture by determining if the patient can fully flatten their hand on a flat surface, was negative. This was due to compensatory MP joint hyperextension. There were no signs of palmar disease.

The fasciectomy was performed under general anesthesia using a pneumatic arm tourniquet and loupe magnification. A Brunner zigzag incision was made, revealing a well-defined pre-tendinous cord over the proximal phalanx with discrete oblique strands extending bilaterally across the PIPJ into the flexor sheath. The cord was fully excised, preserving transverse fibers where possible. Neurovascular bundles were identified and protected throughout. The PIPJ contracture was completely corrected, and perfusion to the finger returned within 30 seconds after tourniquet release, with a capillary refill time (CRT) of less than two seconds. The skin incision was closed with simple interrupted 5-0 nylon sutures.

**Figure 1:**
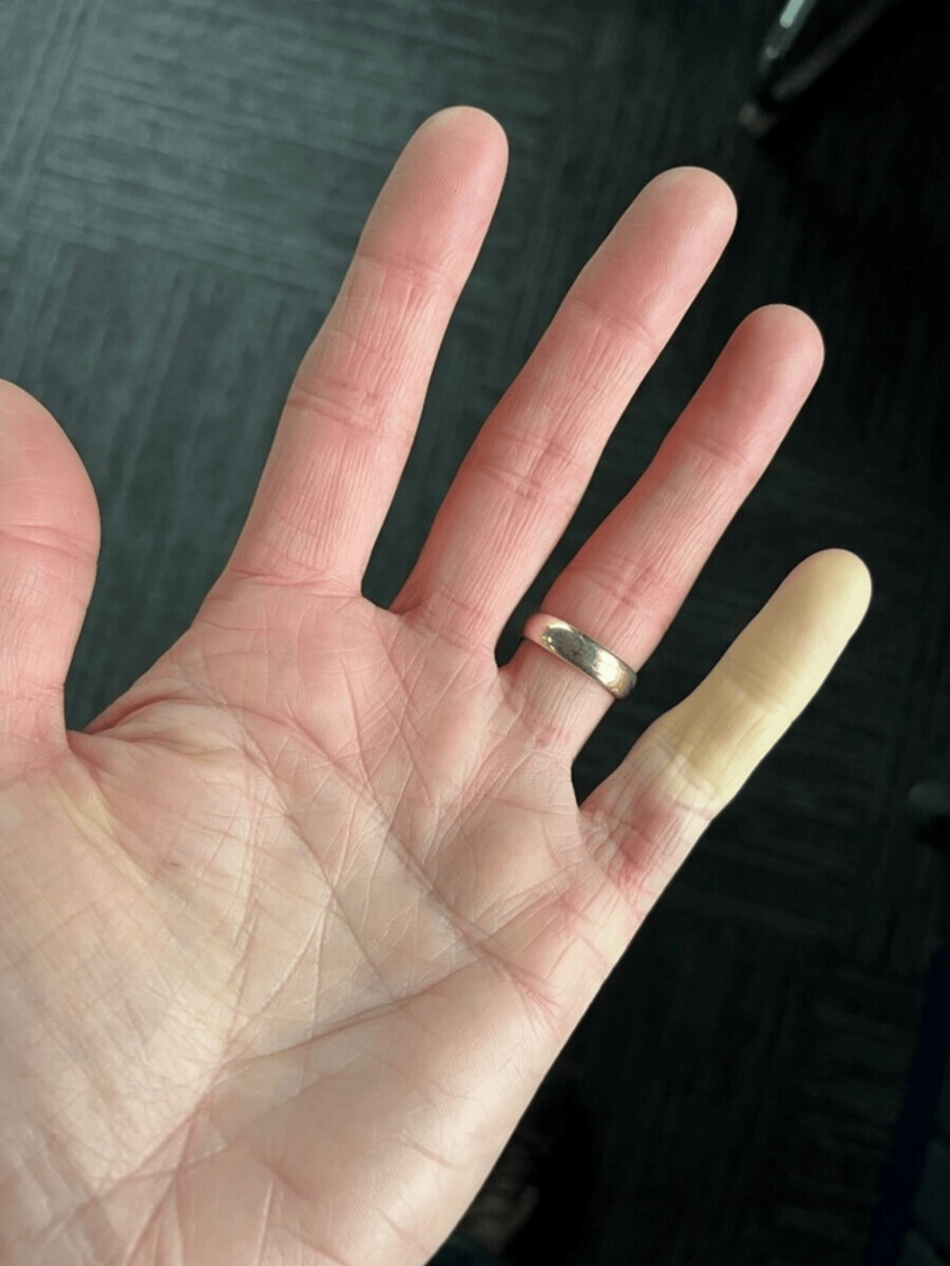
Patient’s own photograph during an episode of cold exposure, demonstrating characteristic pallor in the little finger

Six months post-surgery, the patient was satisfied with the outcome but reported that, in the past month, his left little finger became pale after prolonged cold exposure, improving with rewarming (Figure 1). Examination revealed a pink, warm finger with a CRT of under two seconds, similar to the other fingers. However, a digital Allen’s test, which assesses arterial blood flow by sequentially occluding and releasing pressure on the radial and ulnar arteries, showed sluggish circulation in the ulnar digital artery. This was accompanied by a slightly altered sensation on the ulnar side of the finger. A diagnosis of Raynaud’s phenomenon was made, and the patient was advised to avoid cold exposure, which successfully prevented recurrence.

## Discussion

Raynaud’s phenomenon has not previously been reported as a postoperative complication. However, a case report described a similar occurrence in a 57-year-old Japanese man after a collagenase clostridium histiolyticum (CCH) injection, resulting in persistent painful, pale hands following cold exposure for two years [7]. The authors hypothesized that CCH-induced damage to the perineurium in small veins may impair cold-induced vasodilation (CIVD), potentially leading to Raynaud’s phenomenon. We propose that, in the same way, surgical trauma could also alter this vasodilatory response.

Vibration white finger (VWF), a form of secondary Raynaud’s phenomenon, typically arises from prolonged use of vibrating tools or machinery, causing repetitive trauma [6]. A previous study found an association between VWF and Dupuytren’s disease, reporting a 19.9% increase in Dupuytren’s prevalence among men aged 50-85 with VWF, suggesting a plausible etiological relationship between Dupuytren’s and Raynaud’s [6,8].

While acute vascular injury is a recognized complication of fasciectomy [2], delayed vascular problems such as Raynaud’s phenomenon have not been described. The absence of traditional risk factors, such as smoking or prior injury, in our case suggests the surgical trauma to the neurovascular bundles likely disrupted autoregulation, accentuated by reduced circulation in the ulnar digital artery, rendering the patient more susceptible to cold temperatures.

## Conclusions

This is the first reported case of Raynaud’s phenomenon following fasciectomy for Dupuytren’s contracture. We propose that surgical trauma to the digital neurovascular structures may impair CIVD, predisposing patients to vasospasm. This case highlights the importance of postoperative vascular monitoring and clinician awareness of delayed complications. Surgeons should consider discussing the potential for cold-induced vascular dysfunction with patients undergoing fasciectomy, particularly those with existing vascular sensitivity. Early recognition and patient education on cold avoidance can mitigate symptoms and improve postoperative outcomes.
